# Supplementary material for: Calibration of agent based models for monophasic and biphasic tumour growth using approximate Bayesian computation
Source: J Math Biol. 2024 Feb 15;88(3):28. doi: 10.1007/s00285-024-02045-4 (PMC10869399; doi:10.1007/s00285-024-02045-4)
Supplement: Supplementary file 1 — (pdf 3127 KB) [file 285_2024_2045_MOESM1_ESM.pdf]

Supplementary Document for Calibration of Agent Based Models for Monophasic and Biphasic Tumour Growth using Approximate Bayesian Computation

Xiaoyu Wang, Adrianne L. Jenner, Robert Salomone, David J. Warne and Christopher Drovandi

Contents

1 Analysis of $d_{\max}$ 2

2 Synthetic datasets 4

3 Posterior for synthetic datasets 5

4 Posterior for breast tumour datasets 12

5 Posterior for ovarian tumour datasets 18

6 Bivariate plots for pancreatic tumour datasets 24

1 Analysis of $d_{\max}$

In this section, we analyze the sensitivity of the model parameter $d_{\max}$ to determine the range of the prior distribution. Figures S1-S2 depicts the distribution of distances d for the population of tumour cells based on the volume of the tumour. The d values obtained are proportional to the volume of the tumour. Thus, as the tumour expands, more cells will be located further from its edge. It appears that d remains between 0 and 30 for ranges of tumour volumes relevant to the *in vivo* datasets. In other words, no cell has a distance greater than about $d = 30$. Hence, we assign a uniform distribution constrained by 0 and 50 as the prior for $d_{\max}$. Figure S3 shows the effect of of different values of $d_{\max}$ on the final tumour volume size with other model parameters are held fixed.

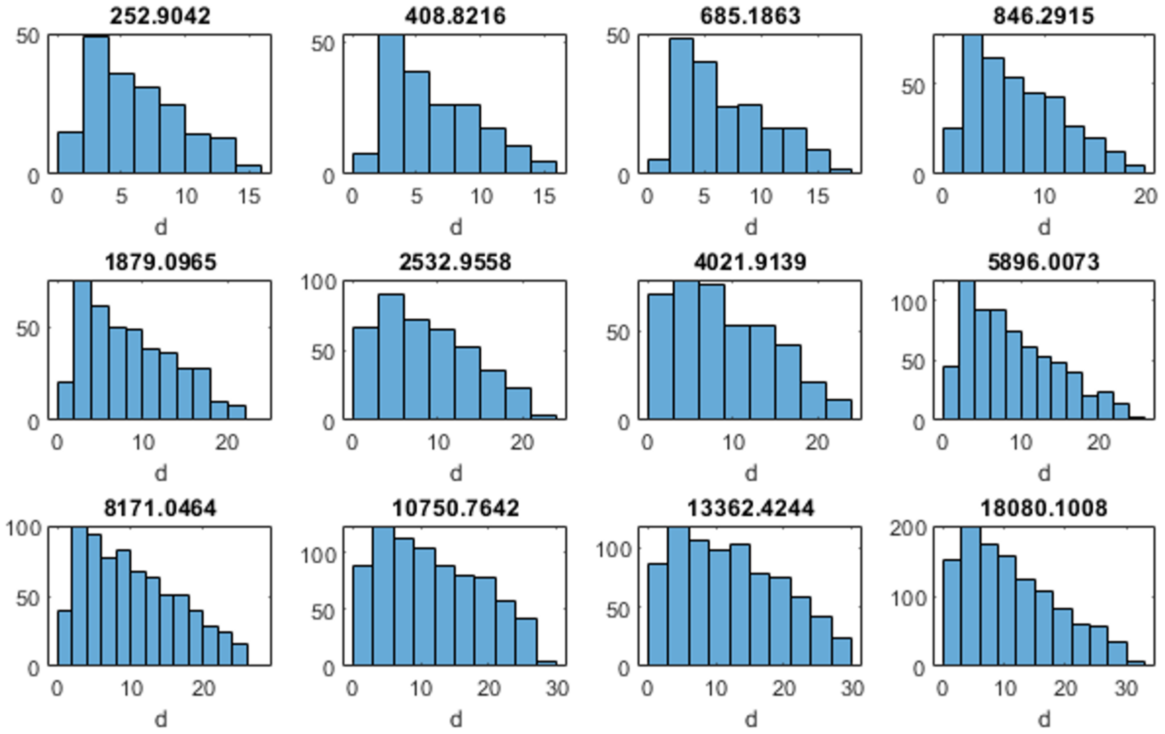

Figure S1: The distribution of distance d for the population of tumour cells based on the volume of the tumour (which is given in the title of each subplot). This is the histograms of d in different number of cells.

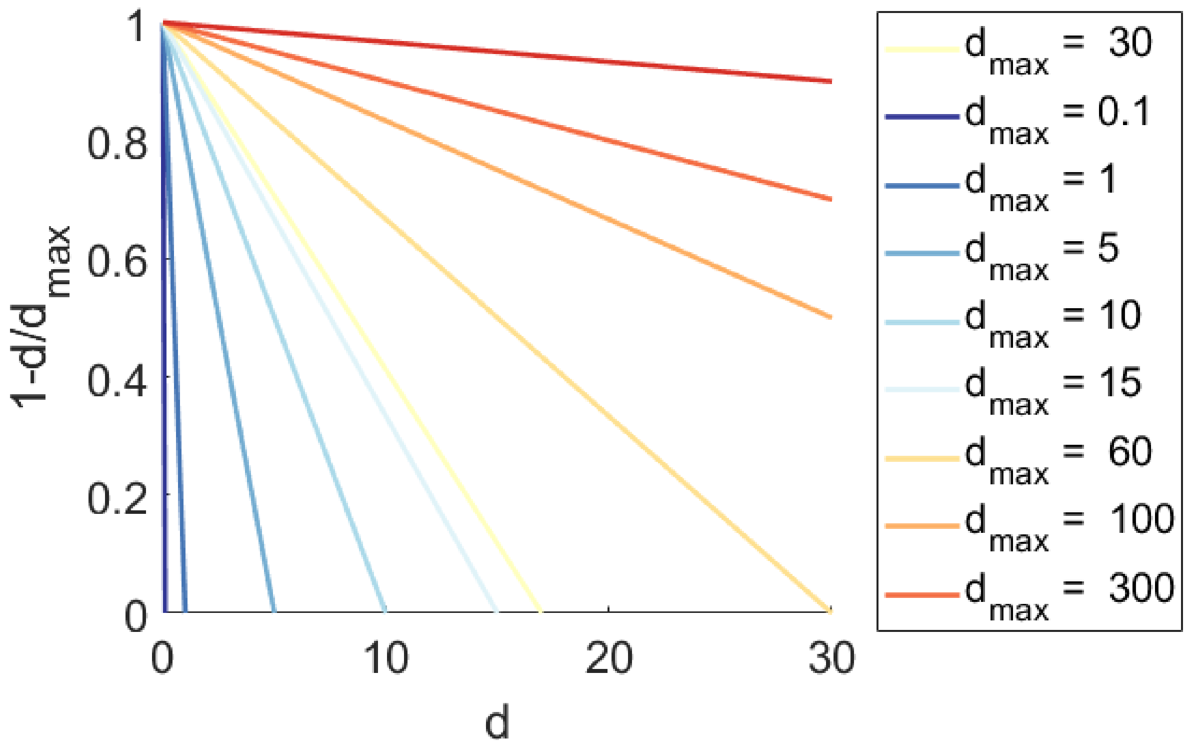

Figure S2: This plot illustrates the relationship between d and $1 - d/d_{\max}$. As $d_{\max}$ increases, the value of $1 - d/d_{\max}$ decays more slowly. It appears that d remains between 0 and 30 for tumor volumes that align with the experimental data, indicating there are no cells with a distance greater than approximately $d = 30$.

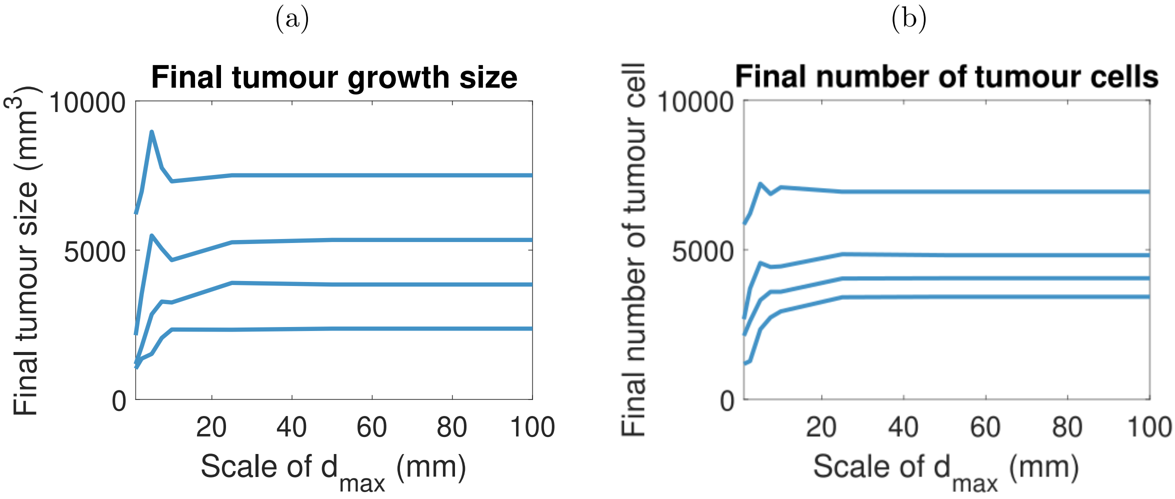

Figure S3: The effect of the value of $d_{\max}$ in VCBM while other model parameters are fixed: (a) final tumour data generated from model for different scale of $d_{\max}$; (b) final total number of cells generated from model for different scale of $d_{\max}$.

2 Synthetic datasets

In this section, we provide the detailed configuration for 5 synthetic datasets in Table S1. The visualizations of each dataset are presented in Figure S4.

Table S1: Parameters used in the generation of the five synthetic datasets. For the first three datasets, we use BVCBM, and for the remaining two, we use VCBM.

Dataset	p_0	p_{psc}	d_{max}	$(g_{\text{age}_1}, g_{\text{age}_2})$	τ	length (days)
1	1	0	17.3	(300, 100)	16	32
2	1	0	17.3	(200,75)	16	32
3	1	0	17.3	(100,300)	16	32
4	0.4	10^{-5}	31	155	N/A	25
5	0.9	10^{-5}	20	171	N/A	66

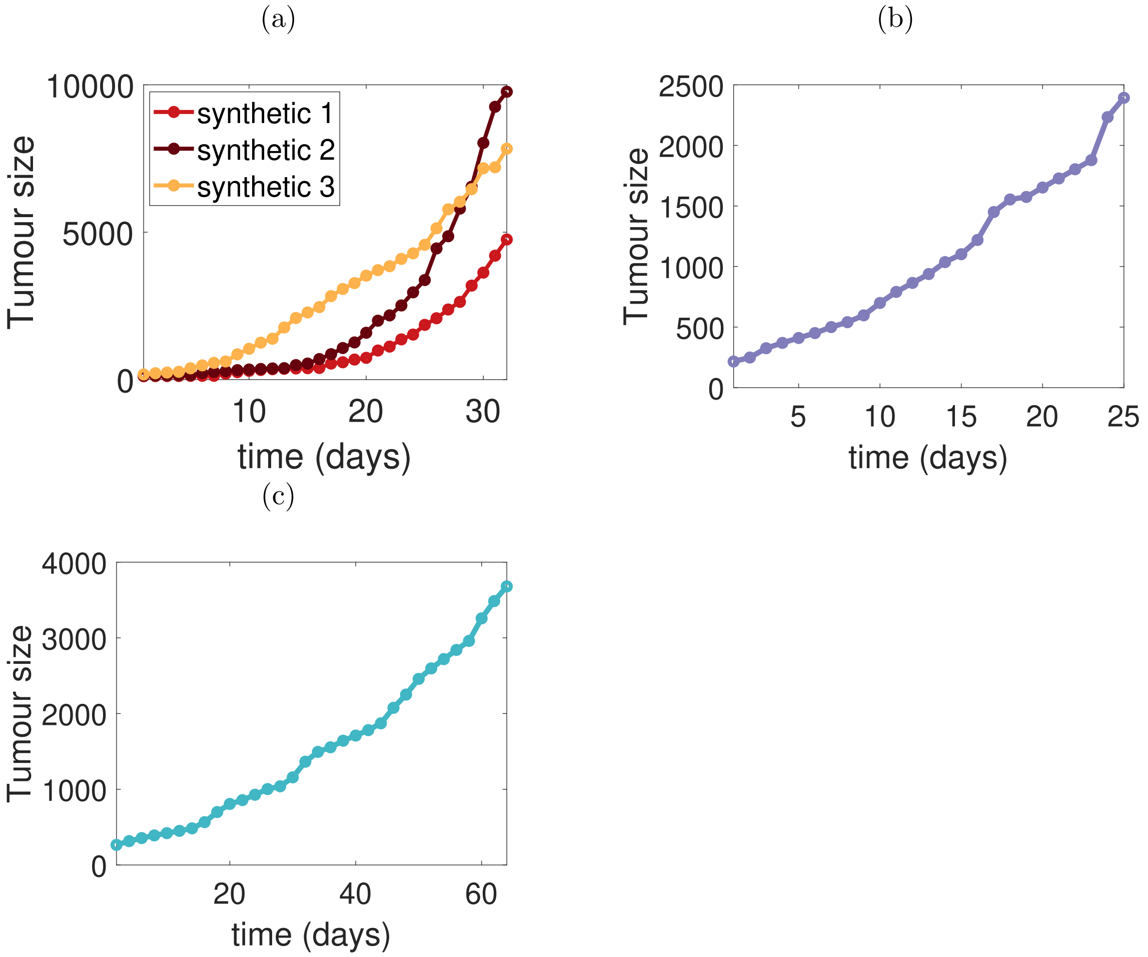

Figure S4: Synthetic tumour volume measurements. (a) shows the synthetic time series datasets 1 to 3; (b) shows the synthetic time series dataset 4 and (c) shows the synthetic time series dataset 5. The parameters used to generate each data set are provided in Table S1. For the first three datasets, we use BVCBM, and for the remaining two, we use VCBM.

3 Posterior for synthetic datasets

In this section, we show the 50%, 80% and 95% posterior predictive intervals. Our results (see Figures S6a, S7a, S8a, S9a and S10a) show that SMC-ABC can recover every synthetic dataset with reasonable accuracy, as the associated tumour volume falls within at least one of the intervals in the posterior predictive plots. The estimated univariate posterior distributions of synthetic time series (see Figures S6-S10) indicate that g_{age} is the most informative parameter for tumour growth in the sense that the posterior is substantially more concentrated compared to the prior. However, the posteriors for p_0 and $d_{\max}$ are not substantially different to the prior, and thus cannot be identified from the data.

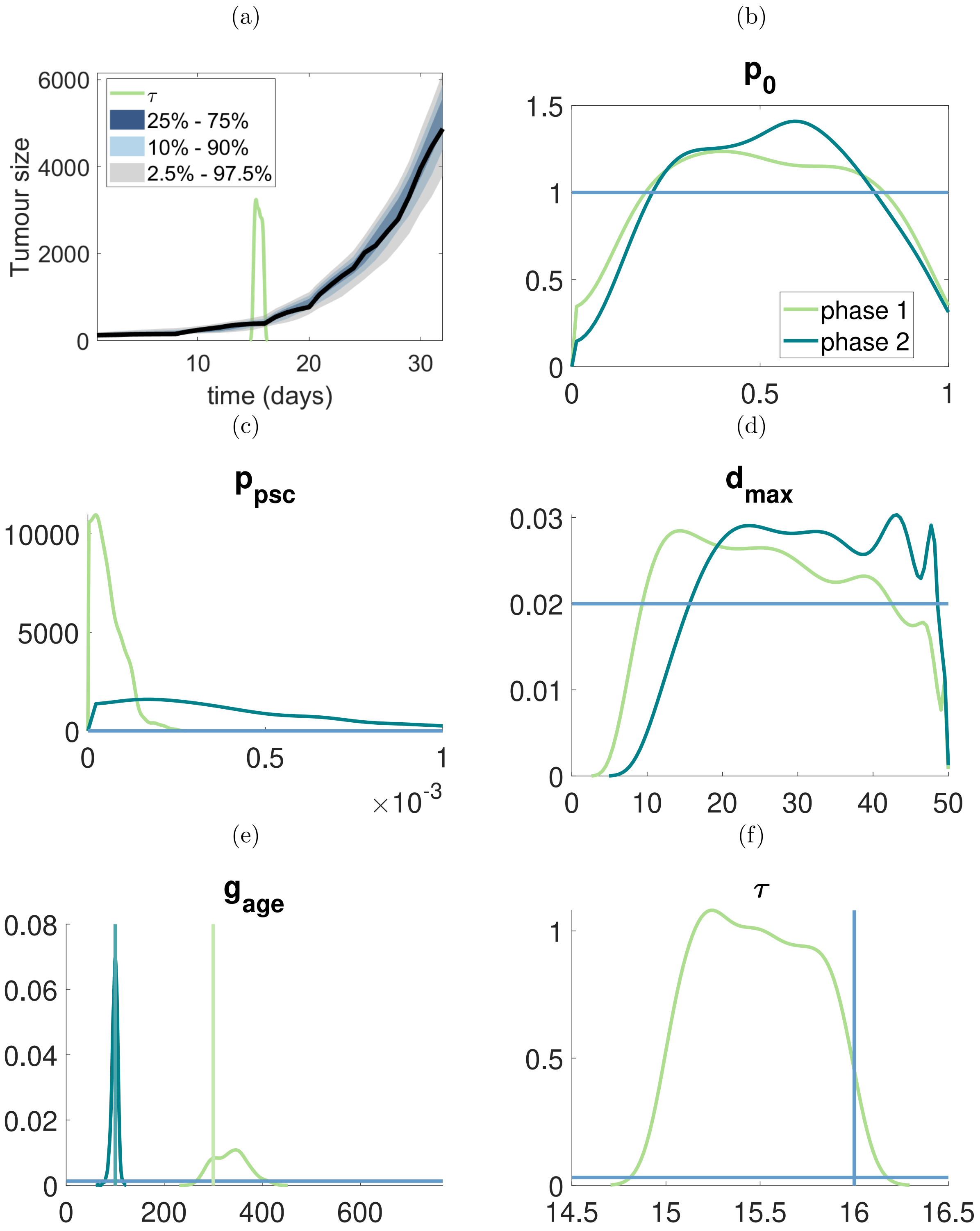

Figure S5: Results for synthetic dataset 1: (a) shows the posterior predictive distribution, the green line shows the scale up posterior distribution for τ used to indicate the switching time of tumour growth, the true density for posterior τ is in (f); (b) - (f) show the marginal posterior for each of parameter, the horizontal blue lines represent the prior distribution and the vertical line in (e) and (f) refers to the "true" values of parameters.

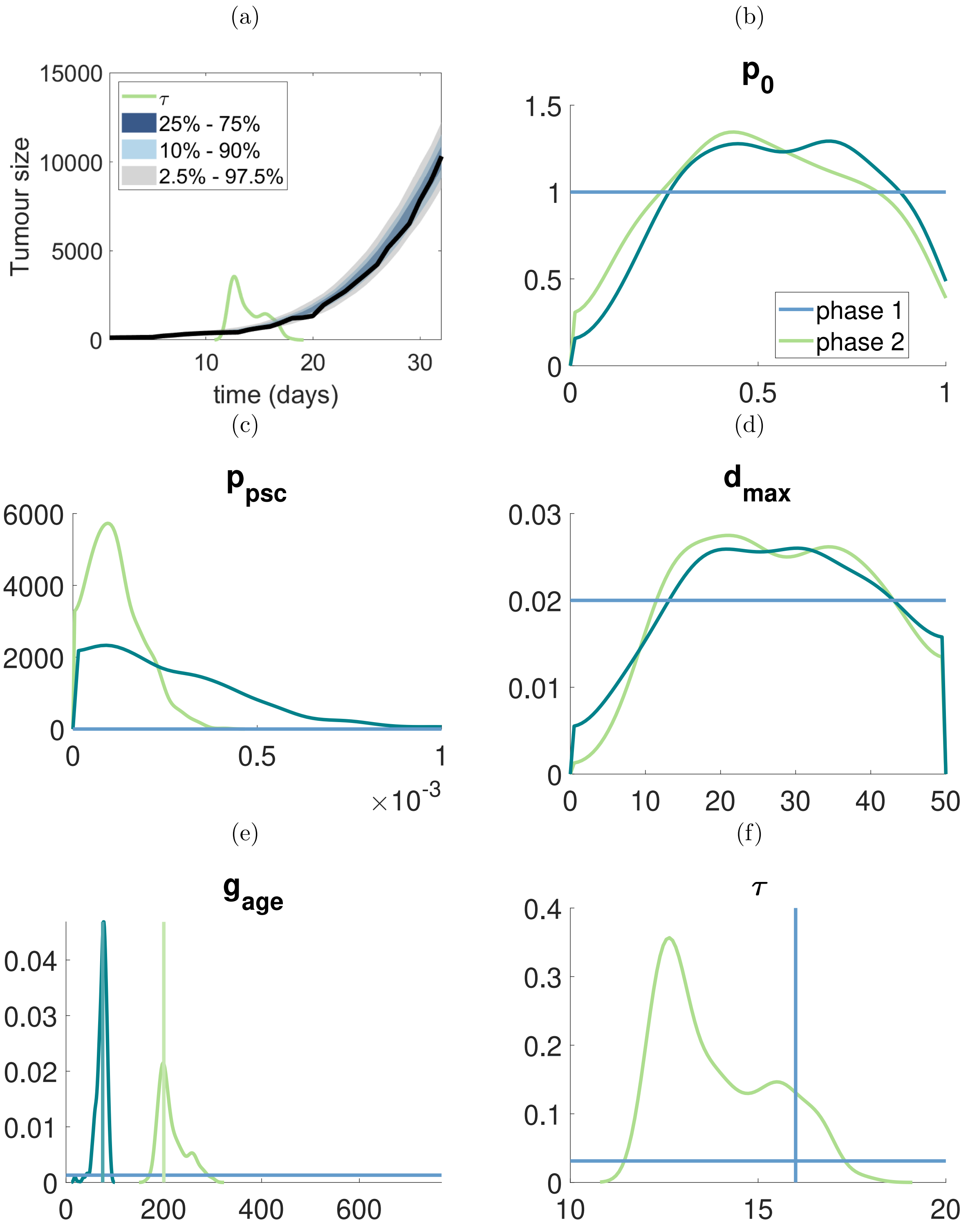

Figure S6: Results for synthetic dataset 2: (a) shows the posterior predictive distribution, the green line shows the scale up posterior distribution for τ used to indicate the switching time of tumour growth, the true density for posterior τ is in (f); (b) - (f) show the marginal posterior for each of parameter, the horizontal blue lines represent the prior distribution and the vertical line in (e) and (f) refers to the "true" values of parameters.

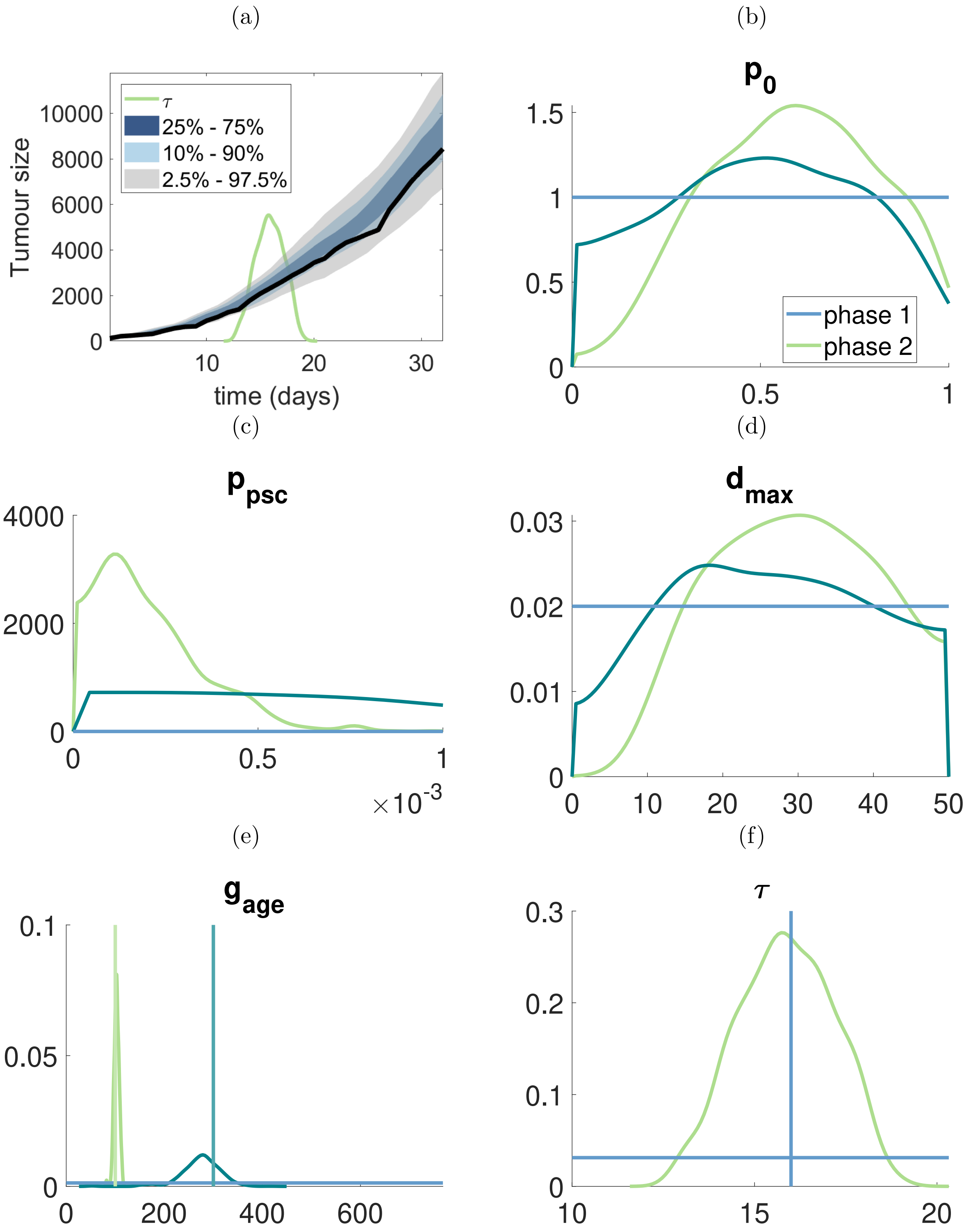

Figure S7: Results for synthetic dataset 3: (a) shows the posterior predictive distribution, the green line shows the scale up posterior distribution for τ used to indicate the switching time of tumour growth, the true density for posterior τ is in (f); (b) - (f) show the marginal posterior for each of parameter, the horizontal blue lines represent the prior distribution and the vertical line in (e) and (f) refers to the "true" values of parameters.

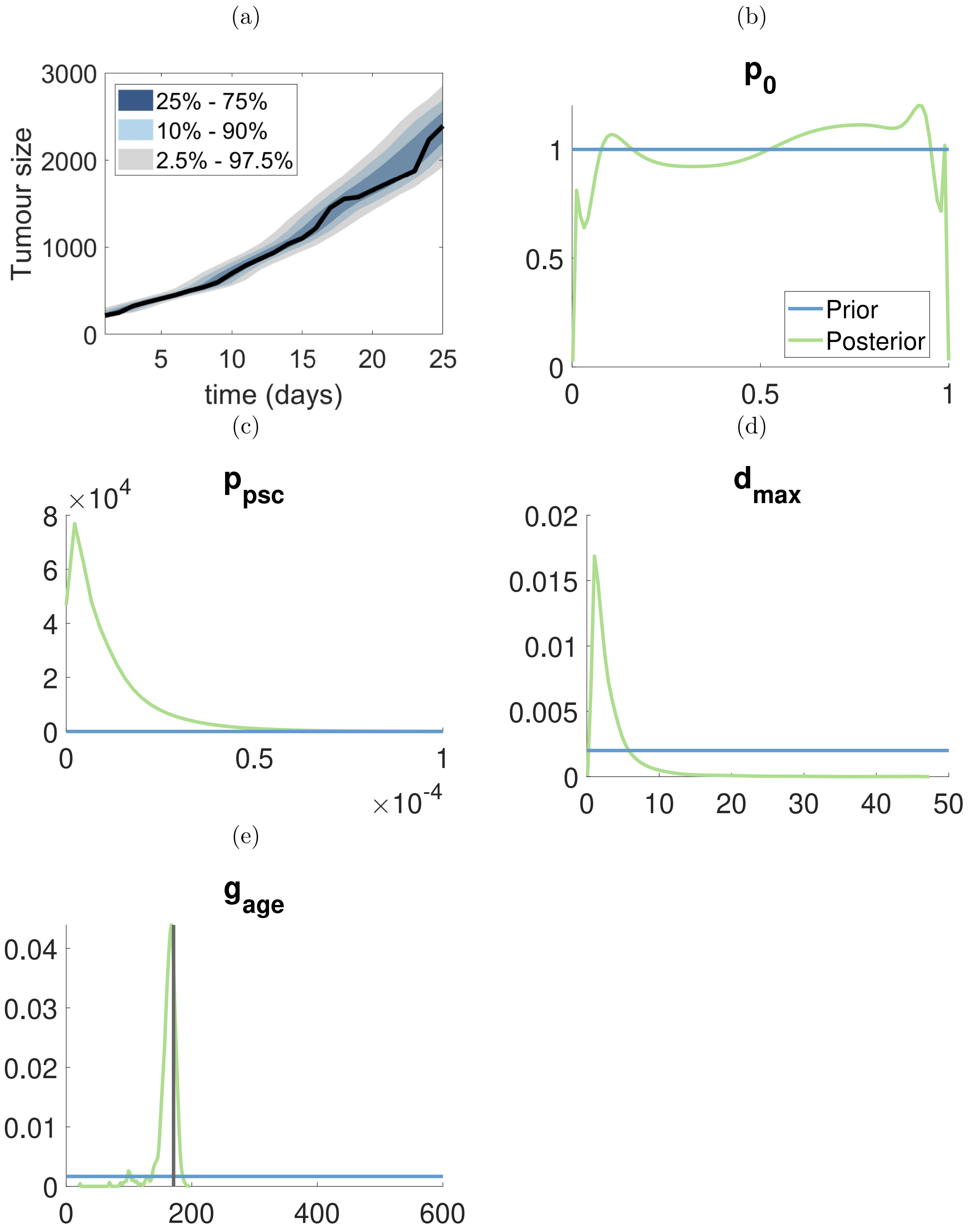

Figure S8: Results for synthetic dataset 4: (a) shows the posterior predictive distribution; (b) - (e) show the marginal posterior for each of parameter, the horizontal blue lines represent the prior distribution and the vertical line in (e) refers to the "true" values of parameters.

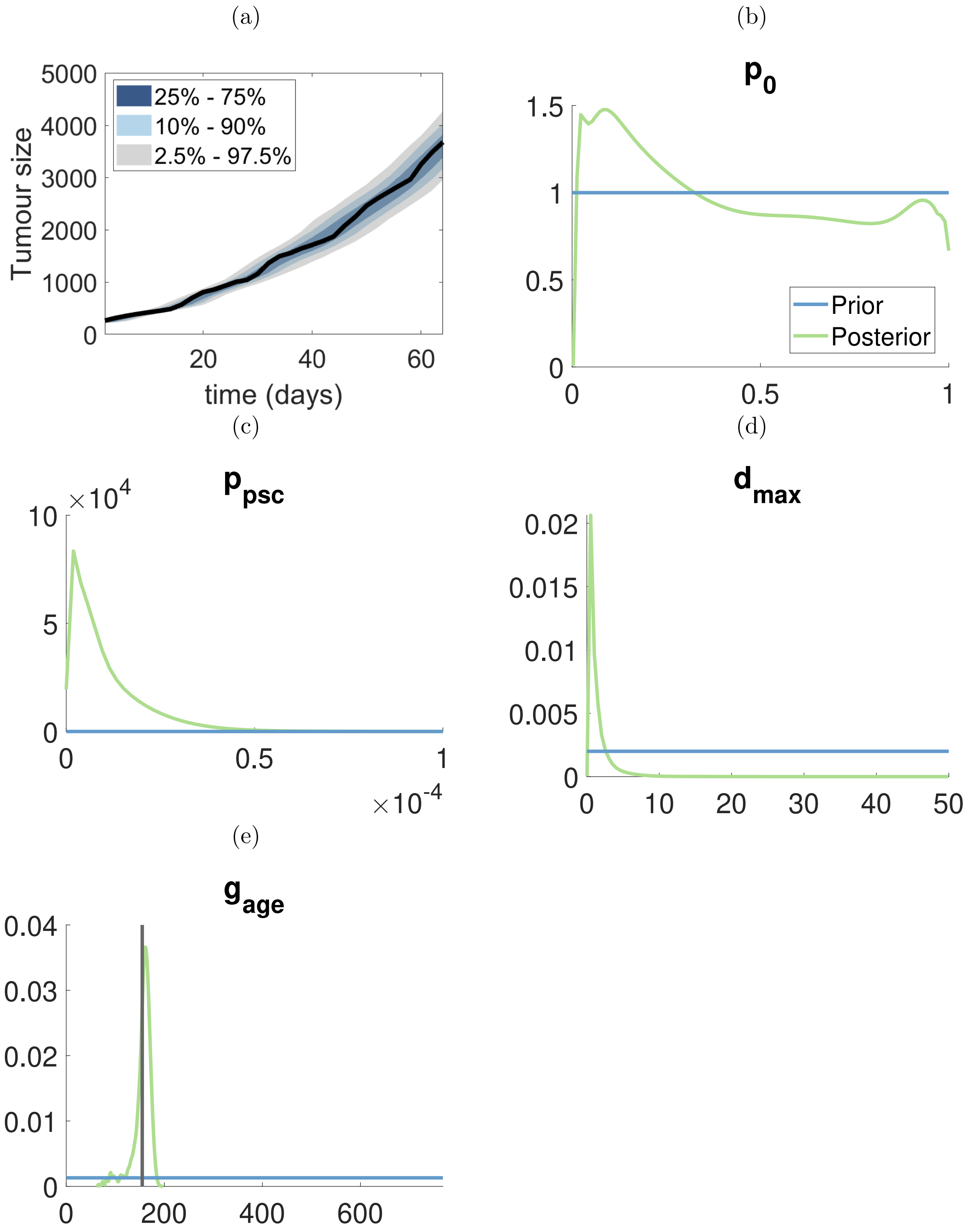

Figure S9: Results for synthetic dataset 5: (a) shows the posterior predictive distribution; (b) - (e) show the marginal posterior for each of parameter, the horizontal blue lines represent the prior distribution and the vertical line in (e) refers to the “true” values of parameters.

Prior Predictive distributions

In this section, we plot the prior predictive distributions by drawing 1000 samples from the prior distribution and then generating simulations from the model. We plot the $(0.25, 0.75)$, $(0.1, 0.9)$, and $(0.025, 0.975)$ prior predictive intervals. It is evident that most of the experimental datasets lie within the $(0.1, 0.9)$ prior predictive interval.

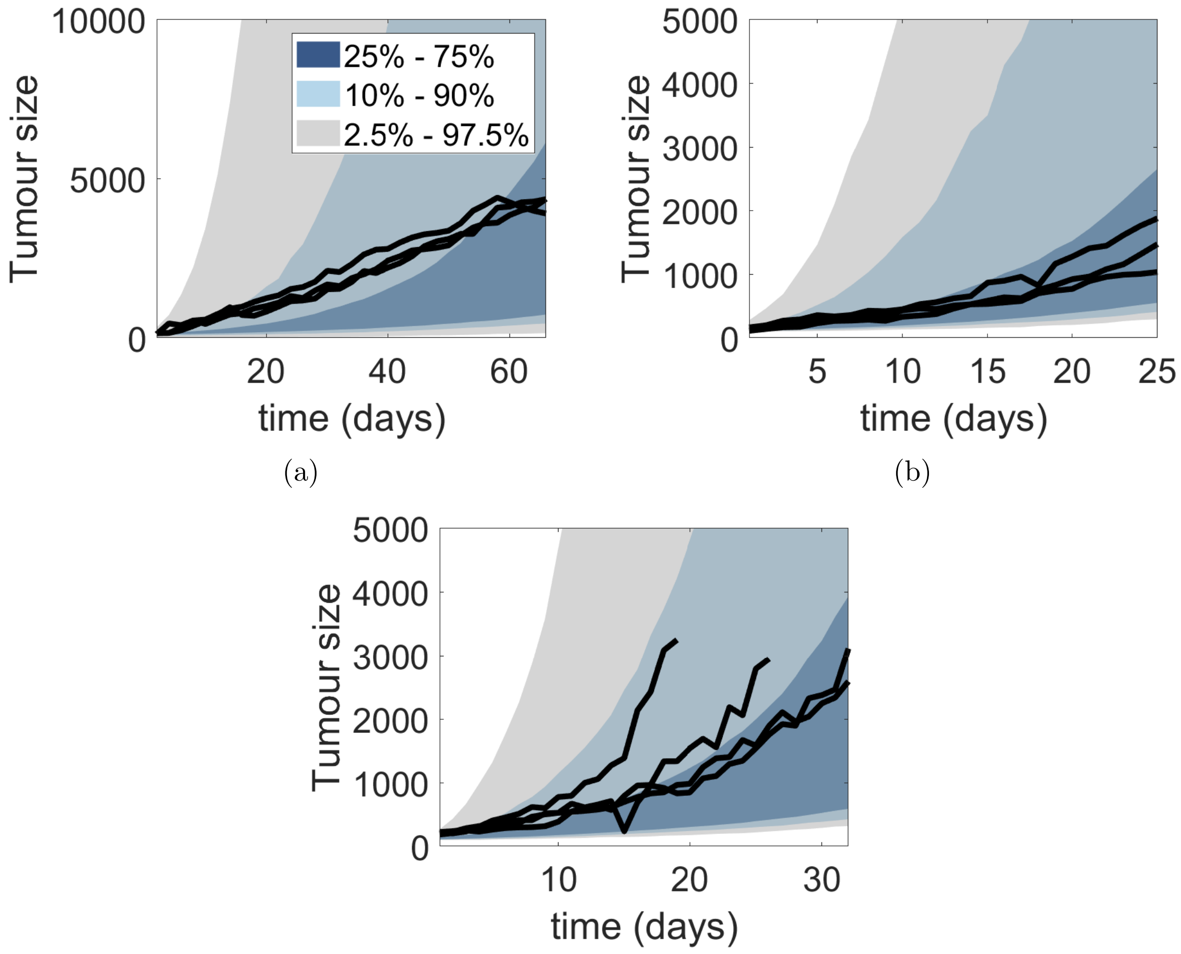

(c)

Figure S10: Prior predictive distributions for each of the experimental datasets. Black solid lines in (a) - (c) are breast, ovarian and pancreatic tumour datasets, respectively. The 50%, 80% and 95% prior predictive intervals are given as shaded regions on the plots, see the legend in (a).

4 Posterior for breast tumour datasets

In this section, we show the univariate and bivariate posterior plots for breast tumour datasets in Figures S11-S16.

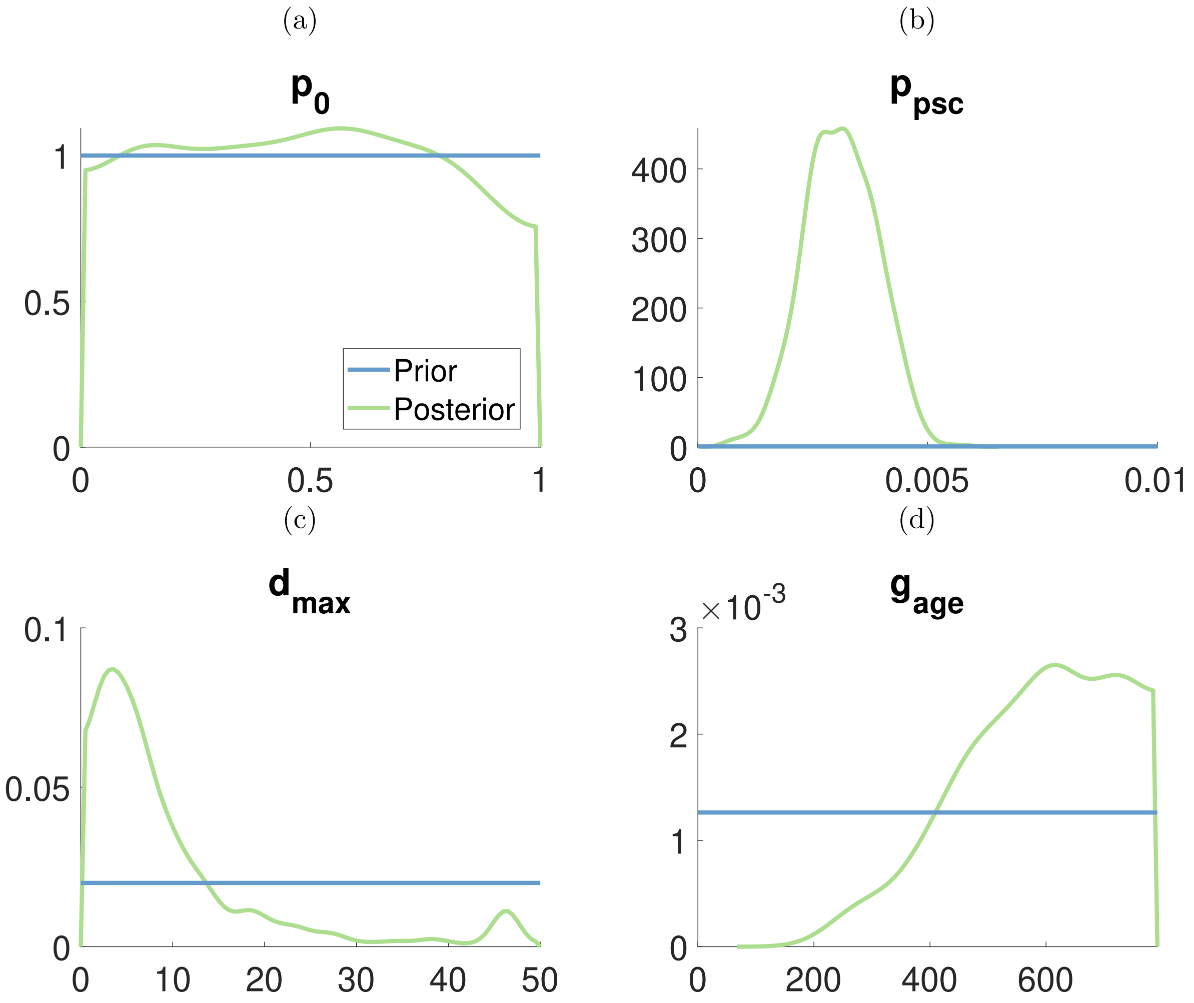

Figure S11: Marginal posterior distributions for first mouse in breast cancer dataset. The horizontal blue lines represent the prior distribution and green lines represent to marginal posterior distributions.

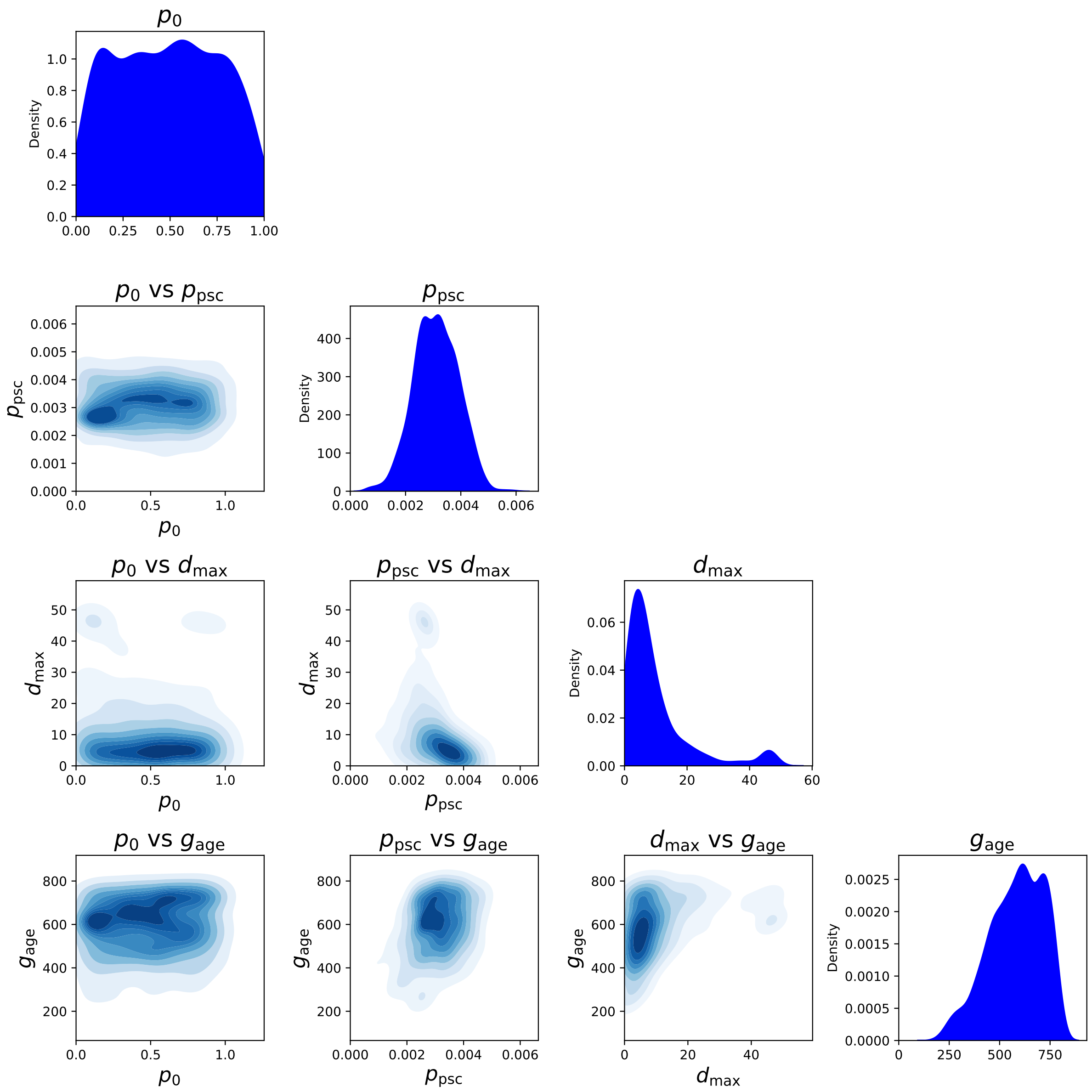

Figure S12: Bivariate plot for first mouse in breast cancer dataset. The diagonal shows the marginal posterior distribution for each parameter, while the off-diagonal plots display the bivariate plots for each pair of parameters.

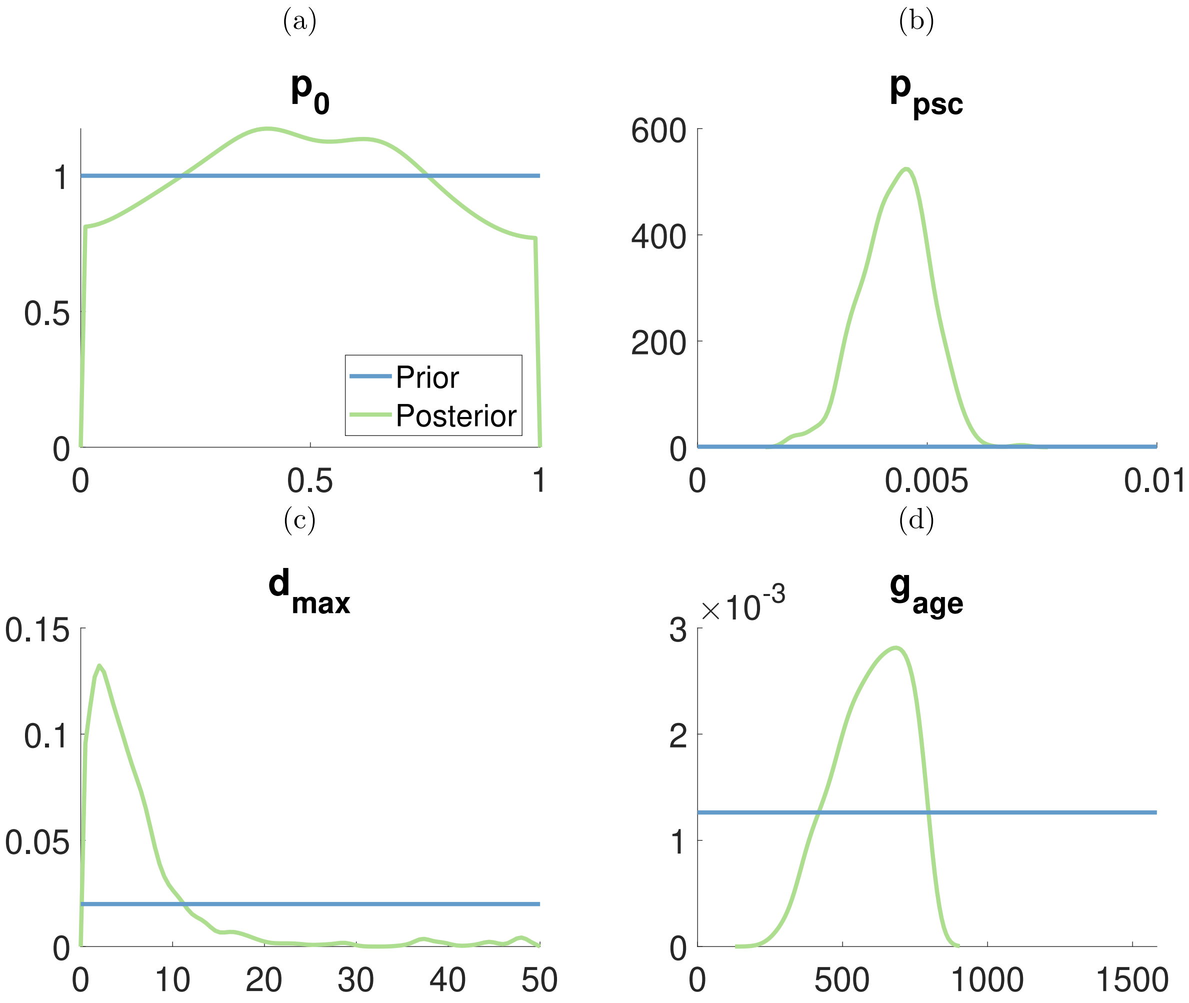

Figure S13: Marginal posterior distributions for second mouse in breast cancer dataset. The horizontal blue lines represent the prior distribution and green lines represent to marginal posterior distributions.

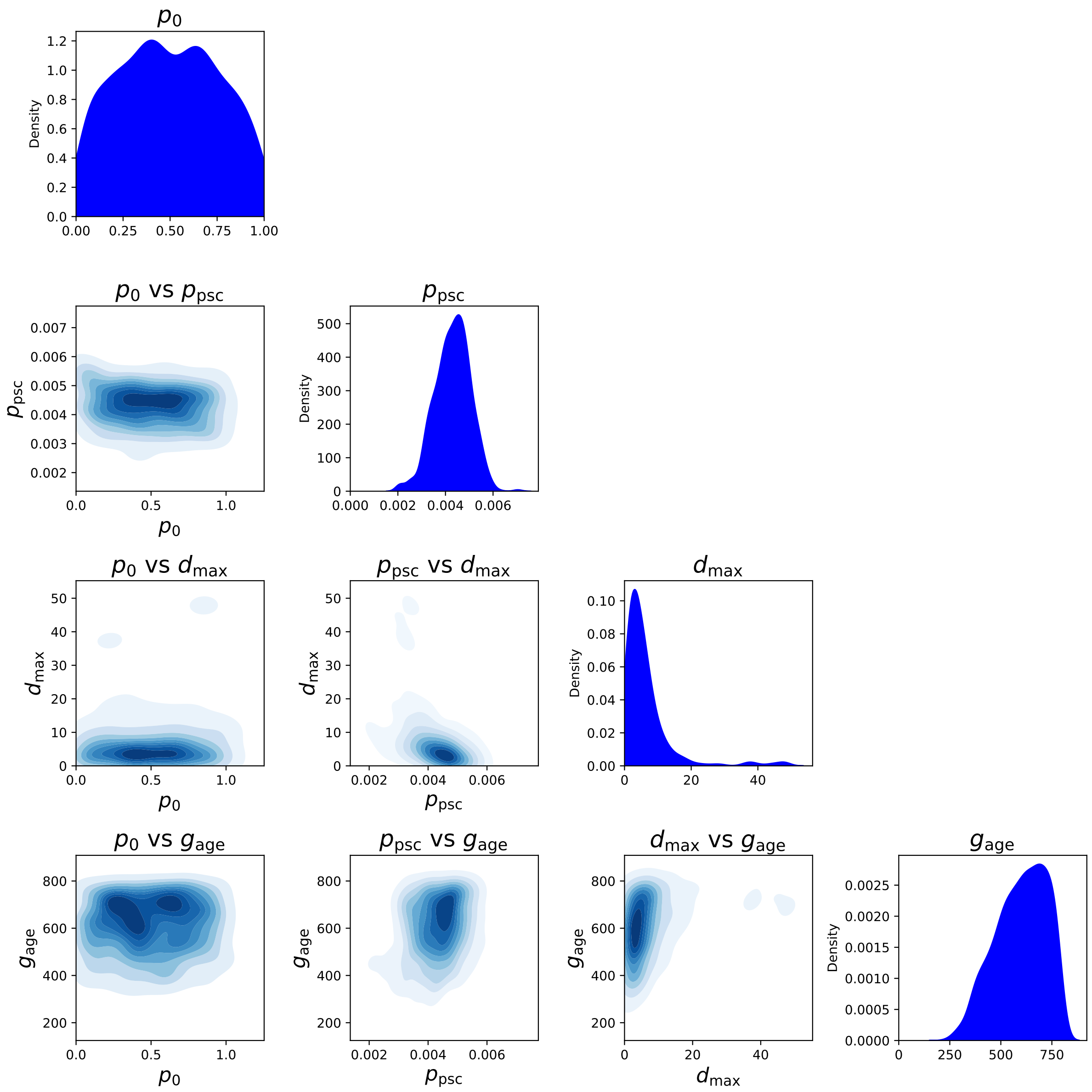

Figure S14: Bivariate plot for second mouse in breast cancer dataset. The diagonal shows the marginal posterior distribution for each parameter, while the off-diagonal plots display the bivariate plots for each pair of parameters.

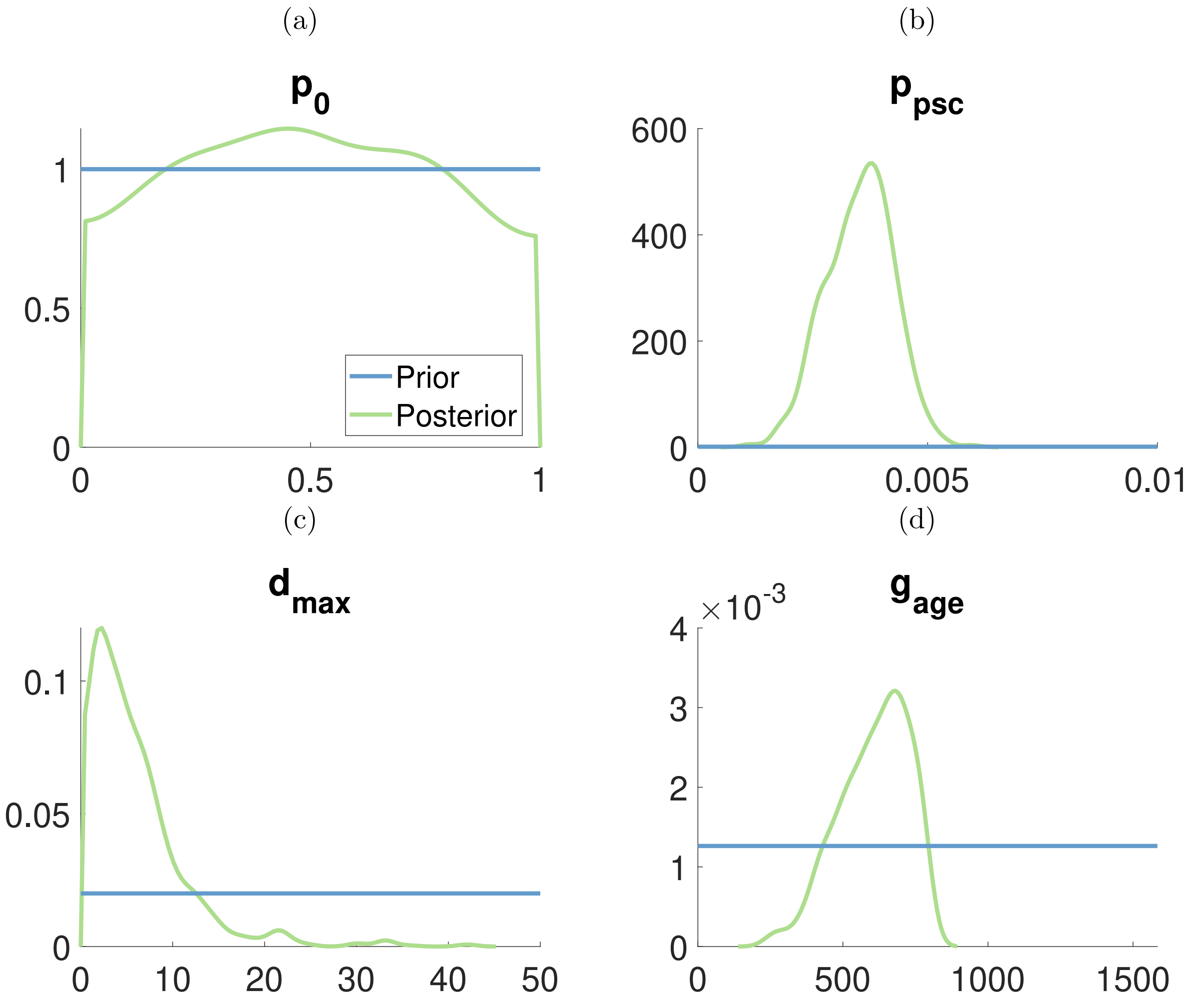

Figure S15: Marginal posterior distributions for third mouse in breast cancer dataset. The horizontal blue lines represent the prior distribution and green lines represent to marginal posterior distributions.

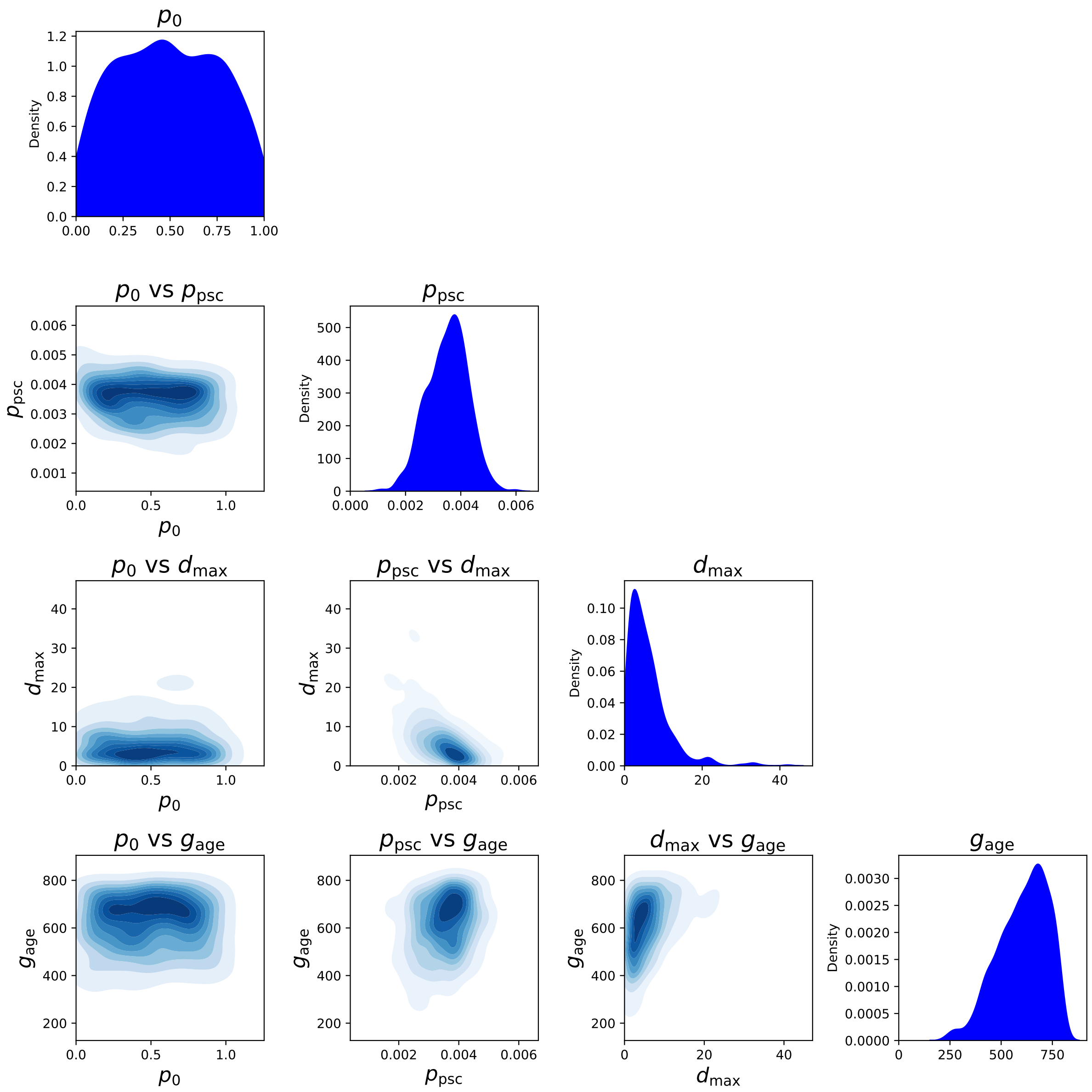

Figure S16: Bivariate plot for third mouse in breast cancer dataset. The diagonal shows the marginal posterior distribution for each parameter, while the off-diagonal plots display the bivariate plots for each pair of parameters.

5 Posterior for ovarian tumour datasets

In this section, we show the univariate and bivariate posterior plots for ovarian tumour datasets in Figures S17-S22.

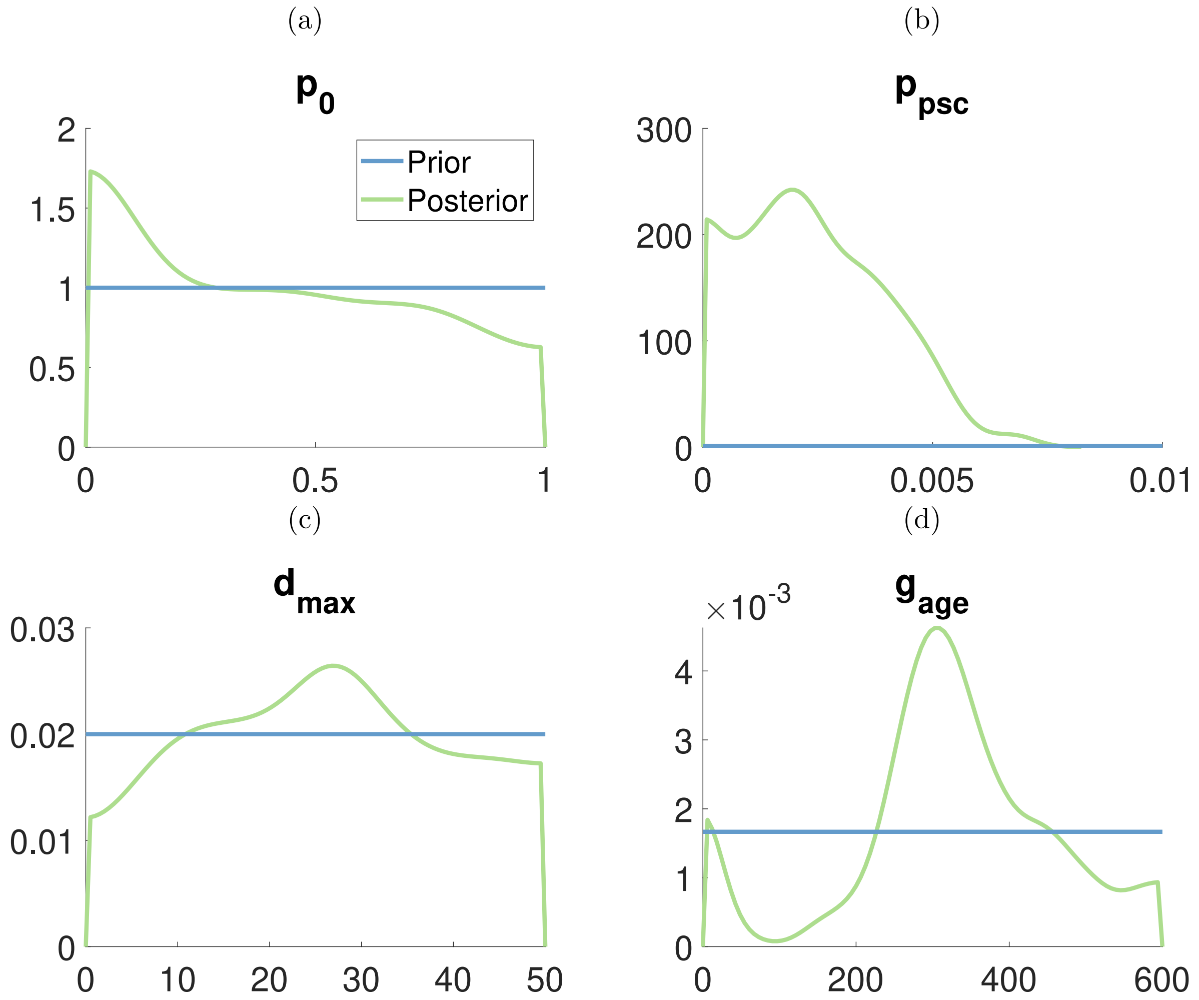

Figure S17: Marginal posterior distributions for first mouse in ovarian cancer dataset. The horizontal blue lines represent the prior distribution and green lines represent to marginal posterior distributions.

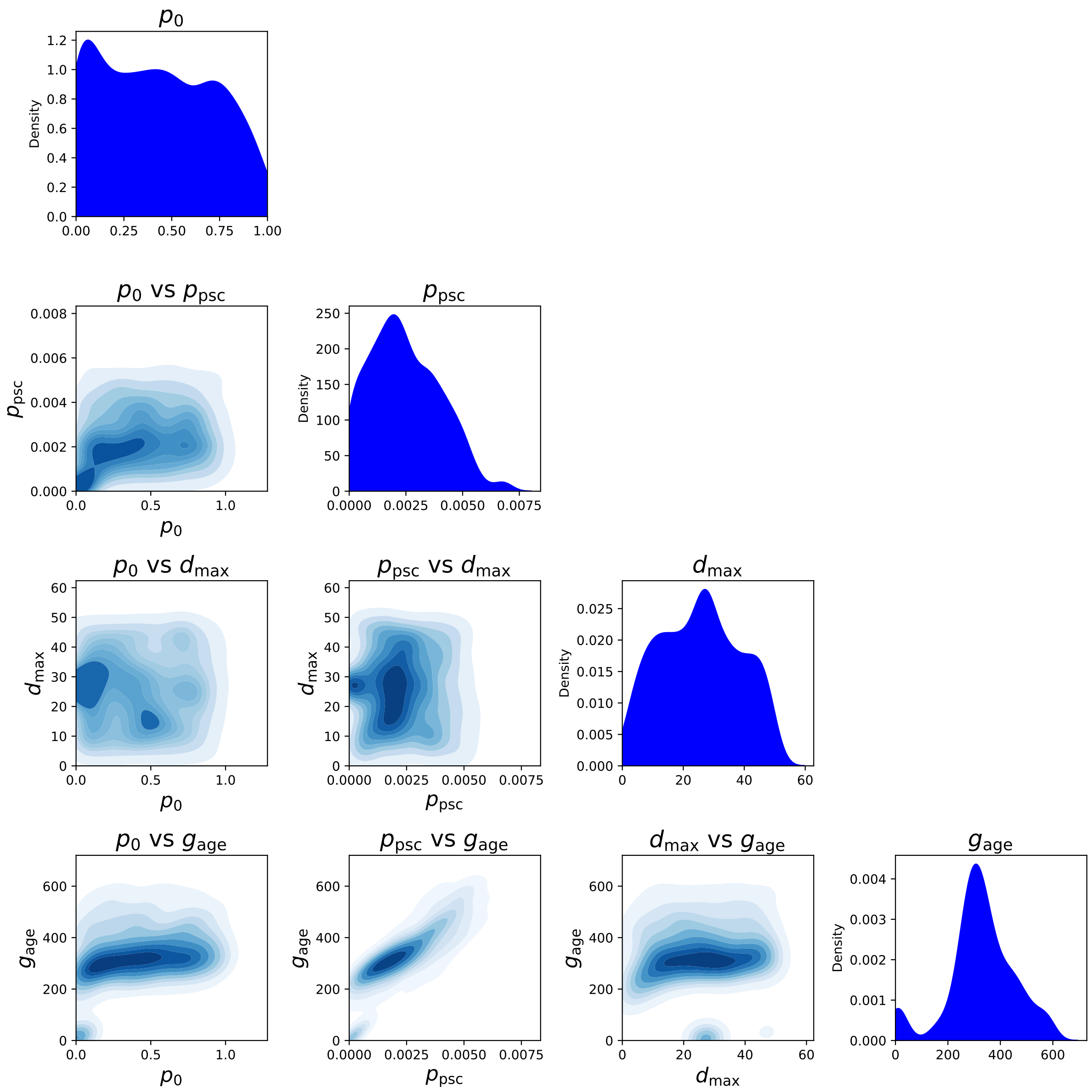

Figure S18: Bivariate plot for first mouse in ovarian cancer dataset. The diagonal shows the marginal posterior distribution for each parameter, while the off-diagonal plots display the bivariate plots for each pair of parameters.

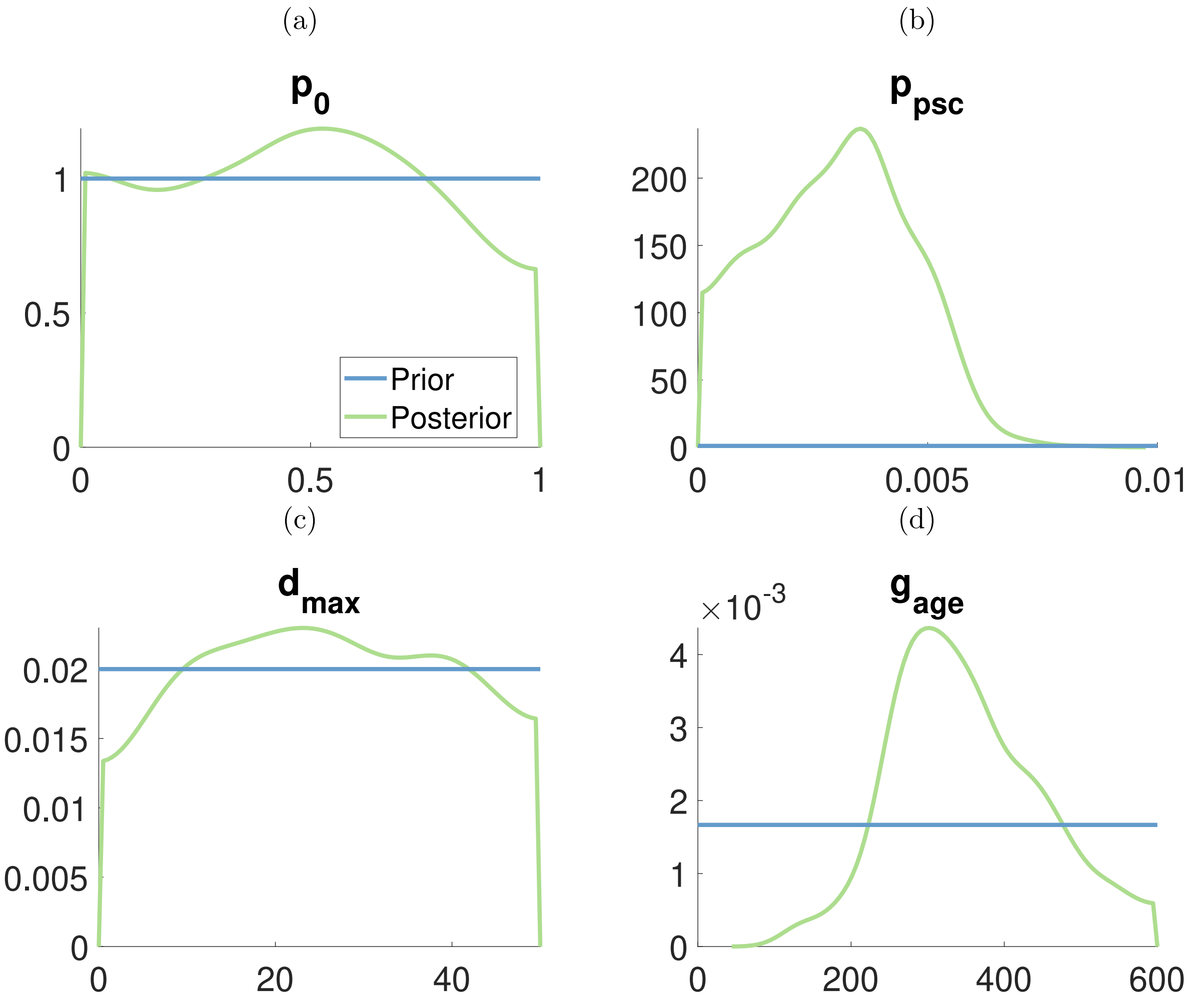

Figure S19: Marginal posterior distributions for second mouse in ovarian cancer dataset. The horizontal blue lines represent the prior distribution and green lines represent to marginal posterior distributions.

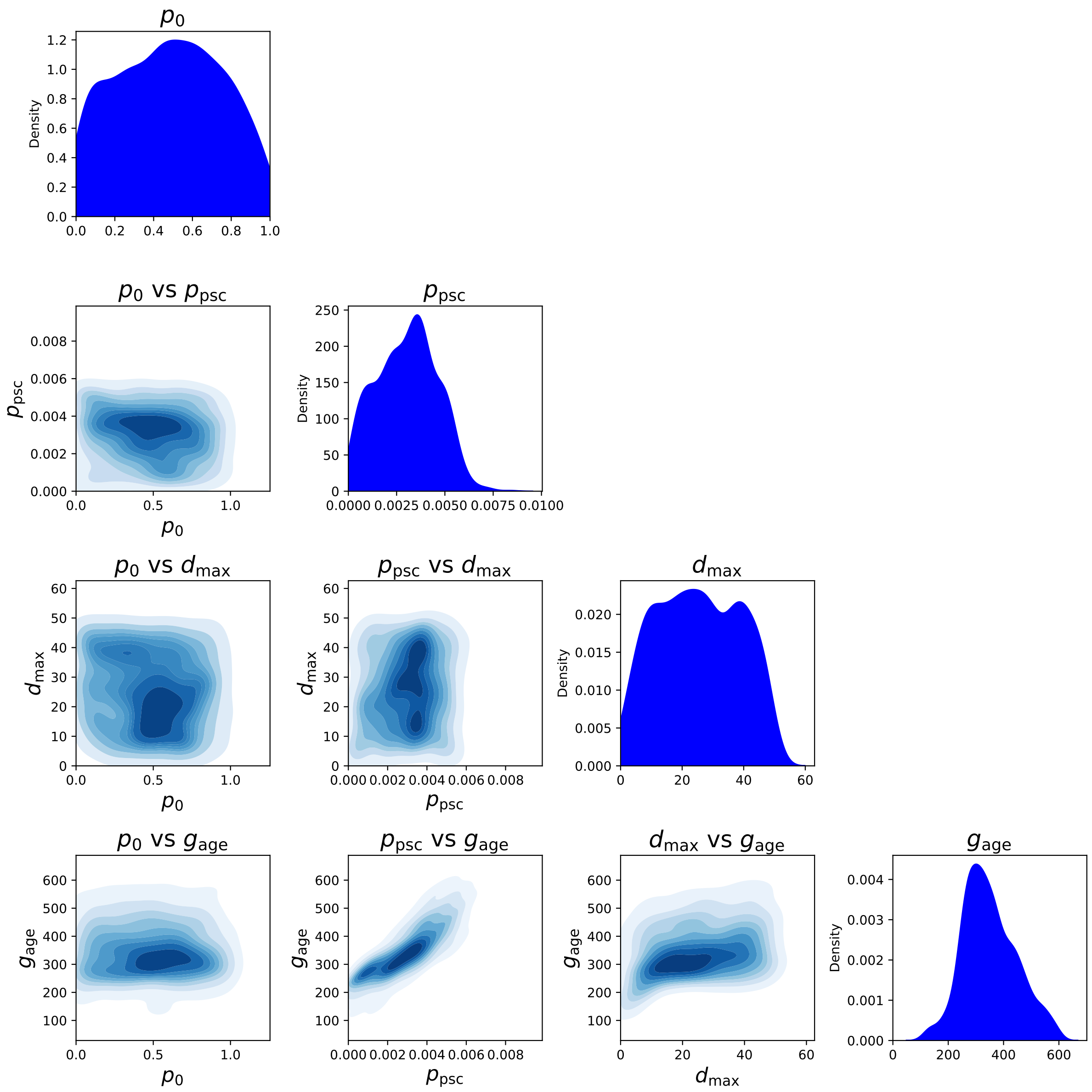

Figure S20: Bivariate plot for second mouse in ovarian cancer dataset. The diagonal shows the marginal posterior distribution for each parameter, while the off-diagonal plots display the bivariate plots for each pair of parameters.

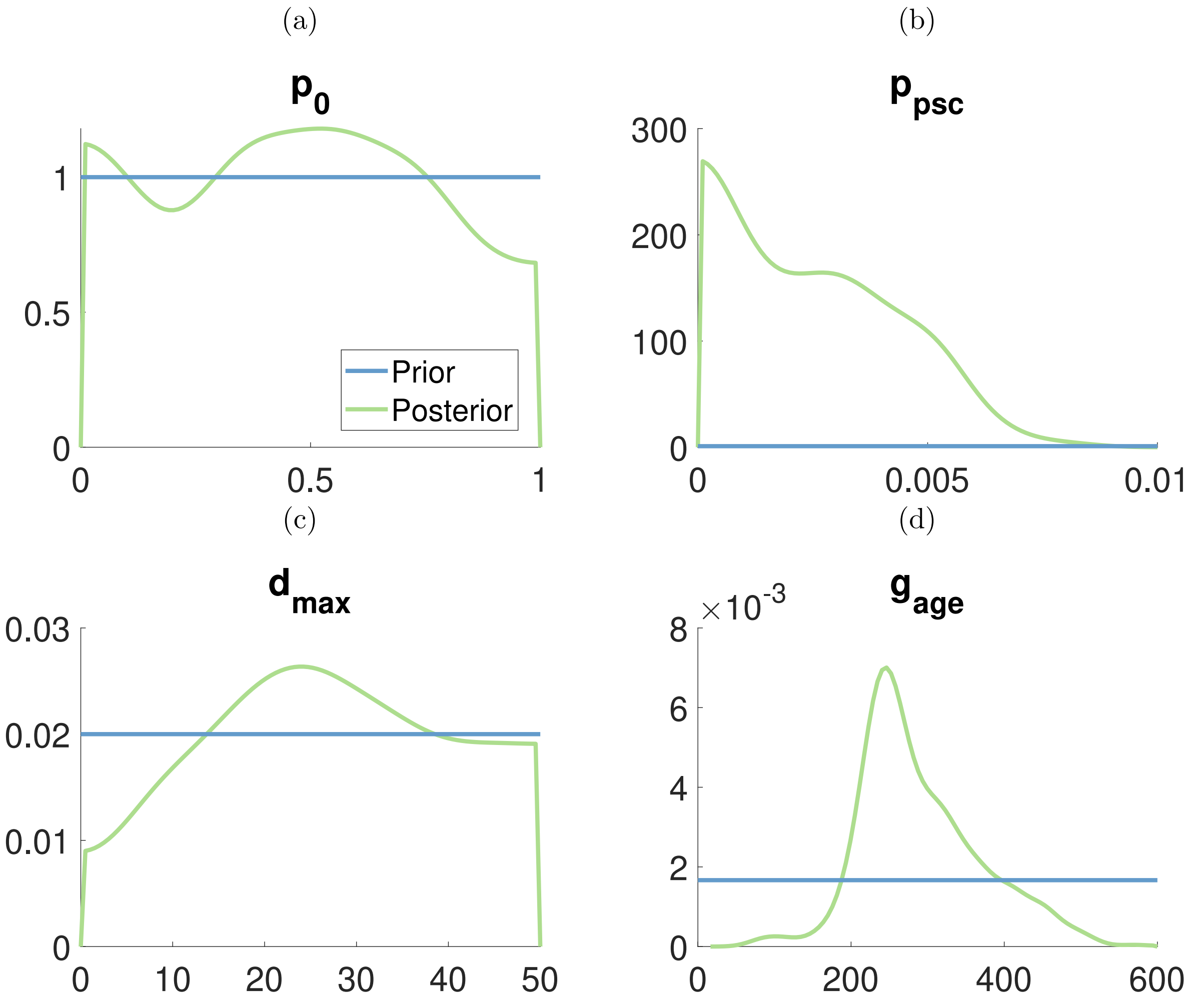

Figure S21: Marginal posterior distributions for third mouse in ovarian cancer dataset. The horizontal blue lines represent the prior distribution and green lines represent to marginal posterior distributions.

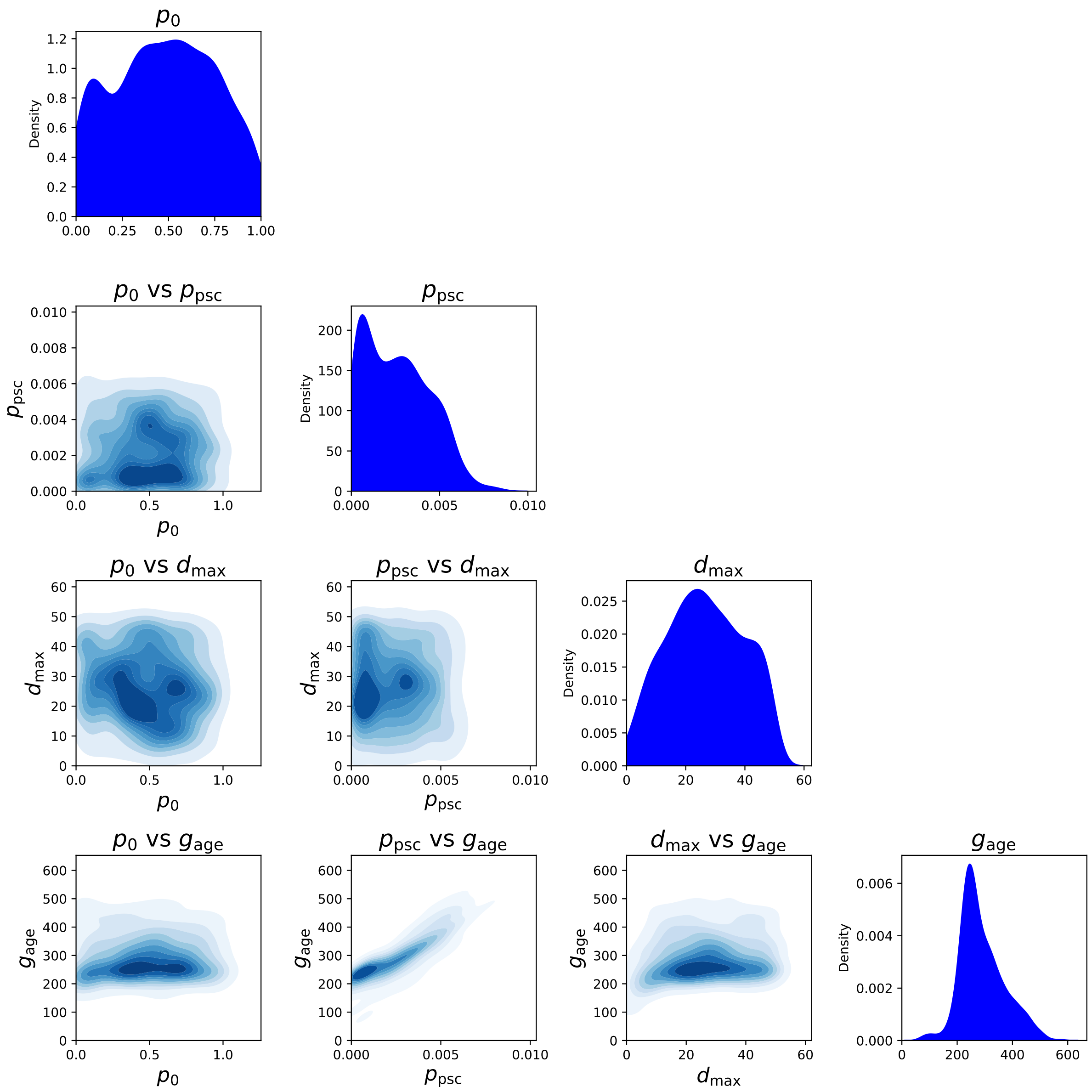

Figure S22: Bivariate plot for third mouse in ovarian cancer dataset. The diagonal shows the marginal posterior distribution for each parameter, while the off-diagonal plots display the bivariate plots for each pair of parameters.

6 Bivariate plots for pancreatic tumour datasets

In this section, we present the bivariate plots for $d_{\max}$ and g_{age} for the two stages, as well as for τ, the switching time, in Figures S23 - S26.

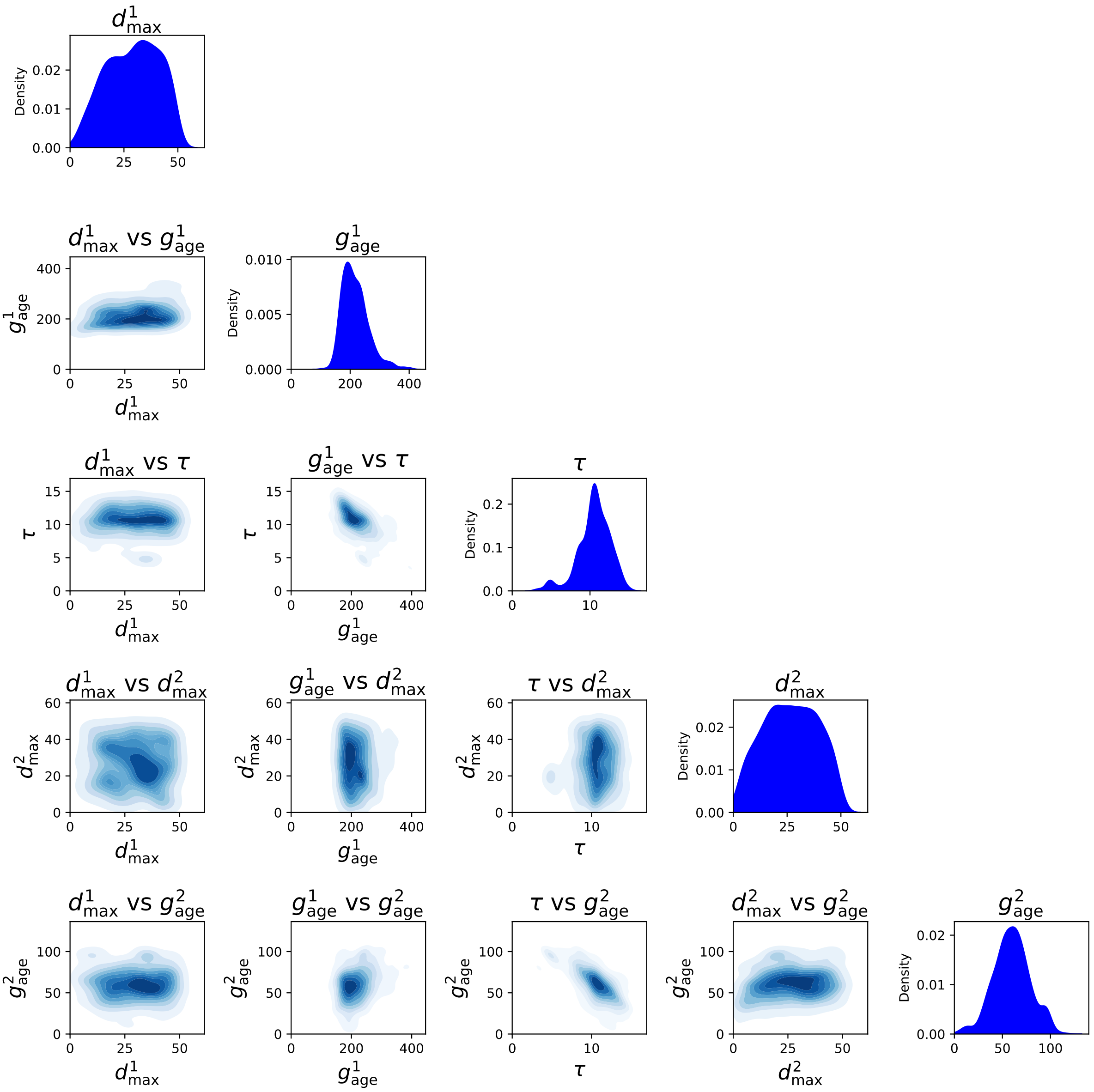

Figure S23: Bivariate plot for first mouse in pancreatic cancer dataset. The diagonal shows the marginal posterior distribution for each parameter, while the off-diagonal plots display the bivariate plots for each pair of parameters.

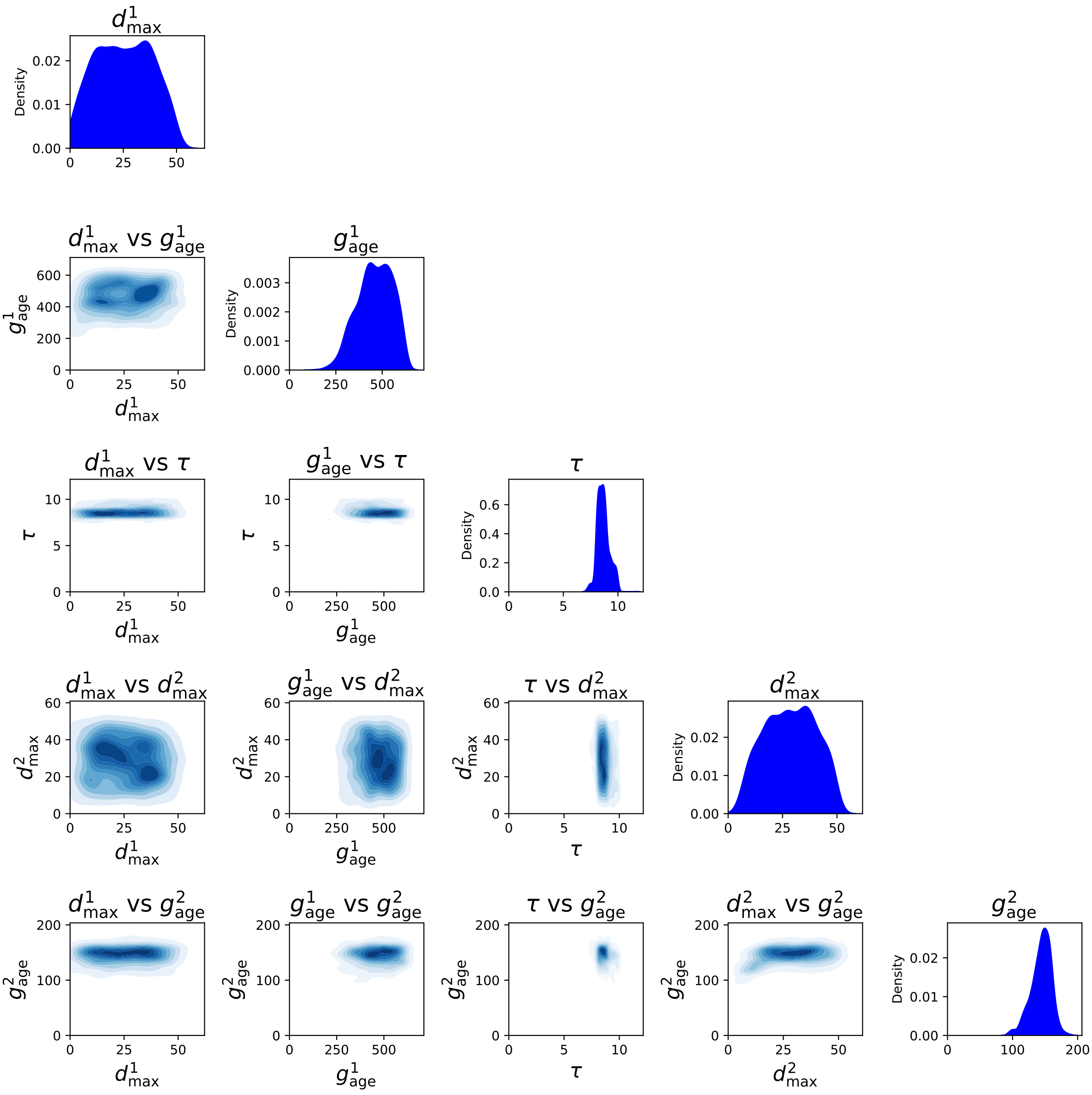

Figure S24: Bivariate plot for second mouse in pancreatic cancer dataset. The diagonal shows the marginal posterior distribution for each parameter, while the off-diagonal plots display the bivariate plots for each pair of parameters.

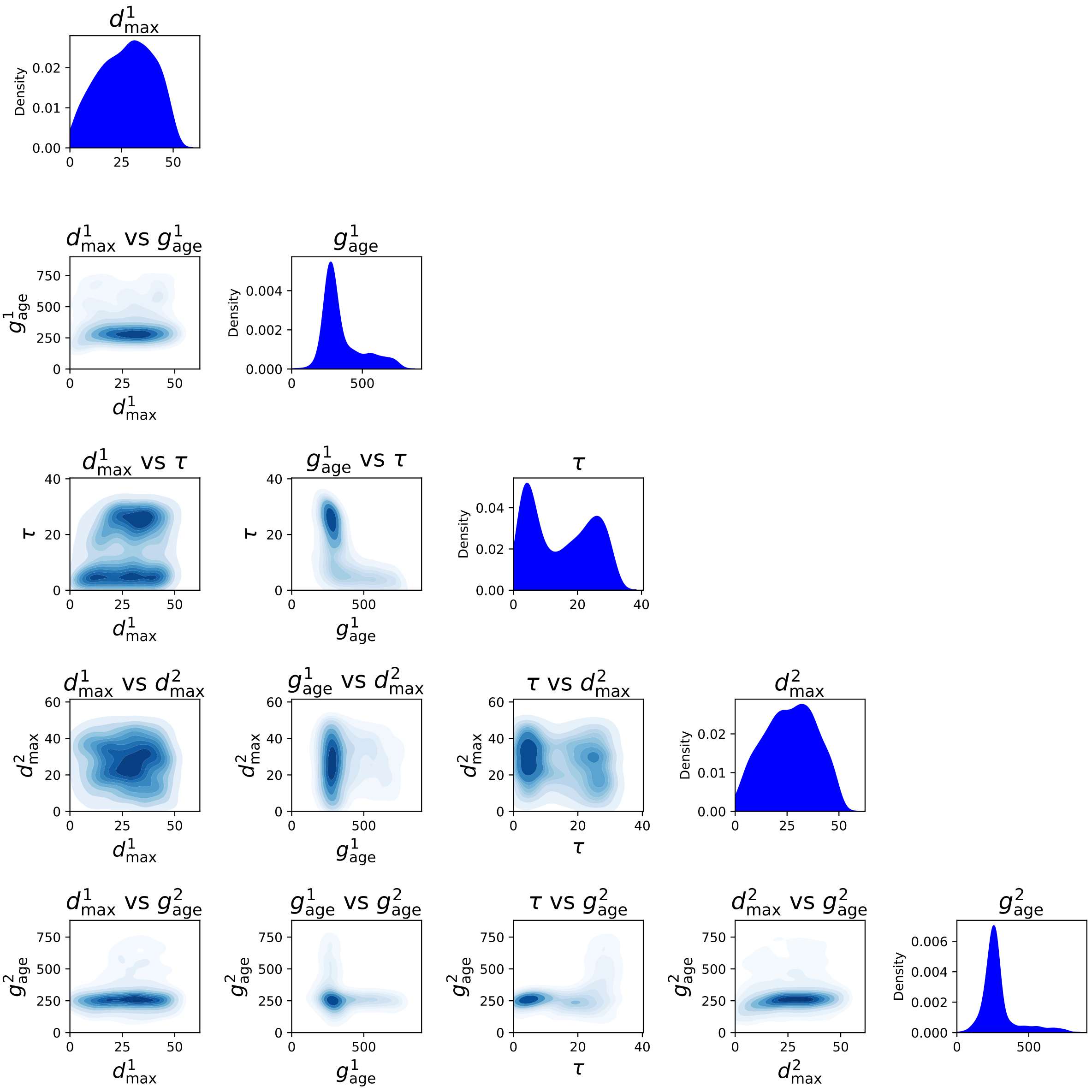

Figure S25: Bivariate plot for third mouse in pancreatic cancer dataset. The diagonal shows the marginal posterior distribution for each parameter, while the off-diagonal plots display the bivariate plots for each pair of parameters.

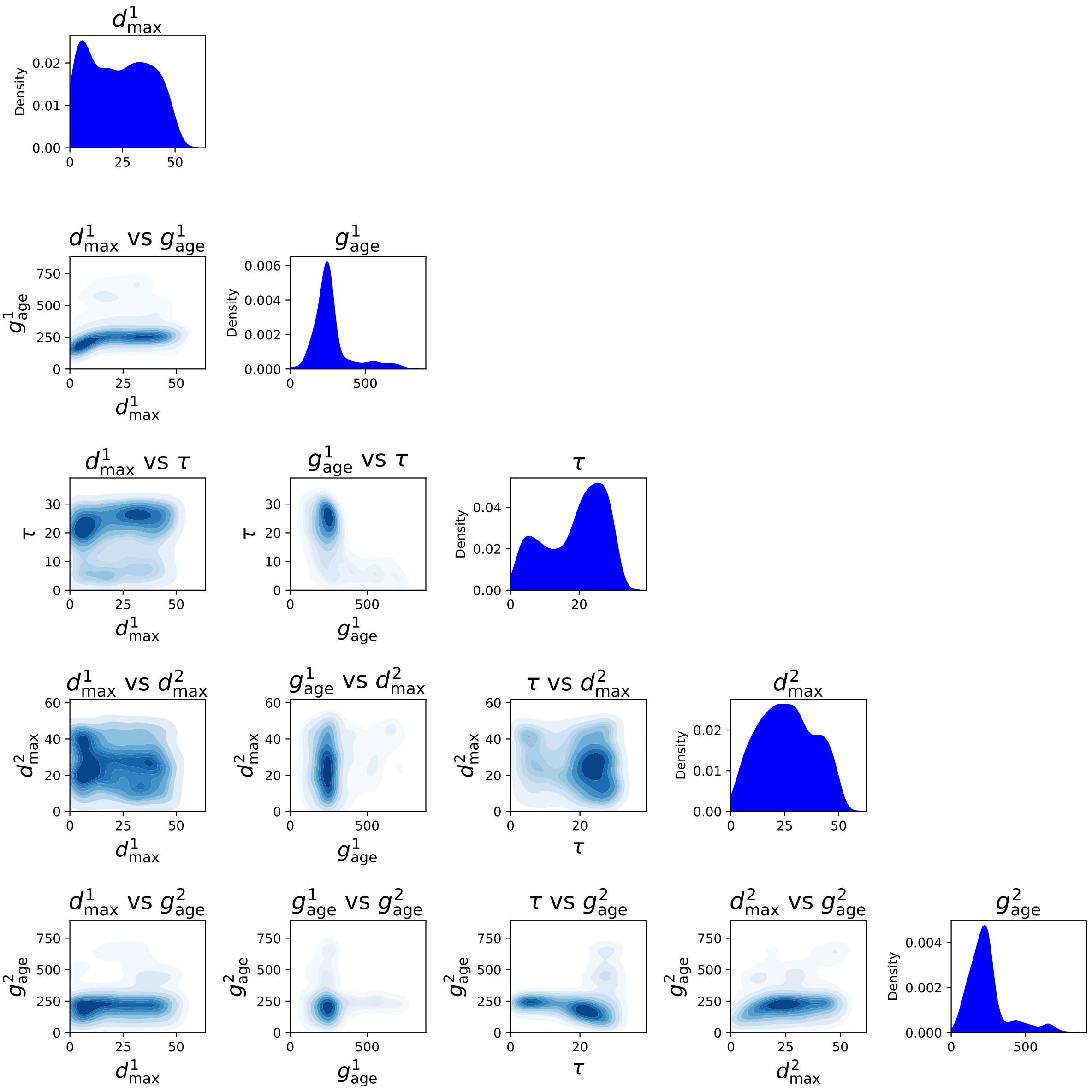

Figure S26: Bivariate plot for fourth mouse in pancreatic cancer dataset. The diagonal shows the marginal posterior distribution for each parameter, while the off-diagonal plots display the bivariate plots for each pair of parameters.
